# Migration-Informed Cancer Equity: A Global Perspective

**DOI:** 10.5334/aogh.5189

**Published:** 2026-05-26

**Authors:** Chul S. Hyun

**Affiliations:** 1 Section of Digestive Diseases, Department of Medicine, Yale School of Medicine, Yale University, New Haven, CT 06510, USA; 2 Yale School of Public Health, Yale University, New Haven, CT 06510, USA

**Keywords:** cancer equity, migration and health, infection-related cancers, gastric cancer prevention, global cancer epidemiology, risk-based prevention

## Abstract

Health equity is now a central aim of modern medicine, yet US cancer-prevention frameworks remain largely shaped by domestic policy histories and nationally bounded conceptions of disparity. While essential for addressing longstanding racial inequities, these frameworks often overlook malignancies driven by infection, geography, and early-life exposure—risks that are well characterized in global cancer epidemiology but imperfectly translated into domestic prevention strategies. As migration reshapes disease distribution, this misalignment leaves persistent gaps in the prevention of cancers such as gastric, liver, and cervical cancer among immigrant and diaspora populations. This perspective advances a migration-informed approach to cancer equity that integrates nativity, early-life exposure, and transnational determinants into prevention design. Using gastric cancer as a case study, we show how omission of migration-linked risk contributes to systematic under-recognition of high-risk populations in low-incidence countries, despite strong global evidence for effective prevention. Achieving meaningful cancer equity in the twenty-first century will require frameworks that extend beyond national borders and reflect how cancer risk is distributed in an increasingly mobile world.

## Introduction

US health-equity frameworks have been shaped by a specific historical mandate: to confront injustice produced within national borders. Emerging from the civil-rights movement and formalized through federal efforts such as the 1985 Heckler Report [1], these approaches have appropriately focused on racial and ethnic inequities rooted in domestic social, economic, and healthcare systems [2, 3]. They remain foundational to public health policy, research priorities, and clinical practice.

More recently, the concept of structural racism has further strengthened this orientation by reframing inequity as a systemic property of institutions rather than individual prejudice. Health outcomes are now understood as the cumulative result of interlocking systems, including housing, education, employment, healthcare, and criminal justice, that differentially distribute risk and opportunity [4, 5]. This framework has been indispensable in clarifying how inequity is reproduced within US society and in motivating reforms across multiple sectors [6, 7].

At the global level, cancer prevention efforts have long shared a concern with inequity and unequal access to health-promoting conditions, but they have emphasized a complementary set of determinants. Initiatives led by the World Health Organization (WHO) and the International Agency for Research on Cancer (IARC) have foregrounded how cancer risk is shaped by geography, early-life conditions, and environmental and infectious exposures, particularly in low- and middle-income settings. This perspective has highlighted malignancies linked to *Helicobacter pylori*, hepatitis B virus, and human papillomavirus as preventable consequences of the place and timing of exposure [8–11]. As a result, global prevention strategies have prioritized population-level interventions such as screening, vaccination, and infection control, reflecting an understanding of cancer not only as a manifestation of social inequity but also as a disease structured by where and when risk is acquired.

As migration reshapes population composition in the United States, the relationship between domestic equity frameworks and these complementary prevention perspectives becomes increasingly consequential. Cancers once concentrated in East Asia, Eastern Europe, or Latin America now persist among immigrant and diaspora populations living in health systems not designed around their exposure histories. Although risk often attenuates after migration, it seldom resets to the level of the host population, creating a pattern in which conventional racial disparities may narrow even as migration-linked risk remains incompletely captured in existing surveillance and prevention frameworks [12, 13].

In the United States, where cancer screening is often opportunistic and fragmented, migration-related risk remains incompletely integrated into prevention strategies. This misalignment highlights a distinct and underappreciated dimension of cancer inequity. When prevention frameworks rely solely on race, current residence, or age, without accounting for early-life exposure and migration, entire populations may fall outside the logic of risk-based prevention. Addressing this gap requires extending equity frameworks beyond their domestic origins and aligning US prevention strategies with a broader understanding of how risk is distributed across populations and over the life course. A migration-informed approach offers a pathway to do so, integrating transnational determinants into the design of modern cancer-prevention systems. Although many migration-associated exposures are acquired early in life and may not be amenable to primary prevention at the time of migration, they remain highly relevant for risk stratification, early detection, and targeted intervention [8–10].

## Migration as a Structural Determinant of Cancer Risk

Cancer risk does not vanish at the border. Migration carries forward early-life exposures, including microbial colonization, environmental carcinogens, and prior access to prevention, that can shape disease risk decades after resettlement. In many cases, these exposures are acquired prior to migration and are not modifiable through primary prevention; however, their identification remains critical for guiding downstream risk stratification and preventive care. These exposure histories then intersect with host-country structures such as insurance coverage, language access, and screening infrastructure, producing patterns of preventable cancer that existing US equity frameworks are not designed to anticipate [12, 13].

Gastric cancer provides one of the clearest illustrations of this dynamic. Although incidence has declined across many high-income nations, several immigrant populations continue to experience risks comparable to those in their countries of origin. Among Asian American populations aged ≥50 years, the incidence of non-cardia gastric adenocarcinoma remains substantially elevated compared with non-Hispanic White populations, even after decades of residence in the United States; this excess is most pronounced among Korean Americans, in whom incidence has been reported to be as high as 14.5-fold [14]. This persistence reflects the durable imprint of early-life exposures, including *H. pylori* infection, together with host, environmental, and health-system factors that shape risk across the life course and may be compounded by delayed recognition and limited access to preventive care in host health systems [6, 8].

Similar migration-linked patterns are observed across other infection-related malignancies. Global analyses demonstrate stabilizing or rising gastric cancer incidence in historically low-incidence countries, driven in part by demographic aging, persistent infection, and migration from high-incidence regions [6, 15]. Parallel trends are evident in hepatocellular carcinoma among foreign-born Asians and Africans, cervical cancer among immigrant women from sub-Saharan Africa and South Asia, and nasopharyngeal carcinoma among Southeast Asian and Pacific Islander diaspora communities [16–19]. Across these diseases, migration structures exposure, susceptibility, and healthcare access in ways that extend across borders and generations.

Despite this evidence, migration remains largely invisible within domestic cancer equity agendas. US surveillance systems such as SEER, NHANES, and BRFSS routinely collect race and ethnicity but seldom capture nativity, age at migration, or parental origin. Cancer registries across North America and Europe similarly aggregate heterogeneous populations under broad categories such as “Asian/Pacific Islander,” obscuring clinically meaningful variation in risk. This structural invisibility delays identification of high-risk subgroups, limits targeted prevention, and reinforces policy blind spots [20, 21].

Taken together, these patterns point to a fundamental limitation of prevailing equity frameworks: risk shaped by migration is neither rare nor incidental, yet it remains systematically underrepresented in prevention design. Treating migration as a structural determinant rather than a demographic descriptor is essential for aligning cancer prevention with the realities of a globally mobile population.

## Gastric Cancer as a Case Study in Misaligned Prevention

Gastric cancer illustrates the consequences of prevention frameworks that do not incorporate migration-informed risk. Although it is among the most preventable malignancies globally [6, 8], it remains largely excluded from national prevention strategies in low-incidence countries, where systems are calibrated to majority-population risk profiles [22, 23]. The result is a persistent paradox: while overall US incidence is low, specific immigrant and minority populations experience burdens comparable to those observed in high-incidence regions yet remain outside organized screening or *H. pylori* eradication programs [23, 24]. Gastric cancer represents a particularly actionable case within this framework; for many other migration-associated malignancies, prevention strategies are more limited, and migration primarily informs risk recognition rather than direct intervention.

This disconnect is reflected in population-level outcomes. In 2025, an estimated 30,300 new gastric cancer cases and 10,780 deaths are expected in the United States [25]. Despite this burden, no federal strategy exists for systematic *H. pylori* detection or eradication. Five-year relative survival is often cited at approximately 37.9%, among the lowest of major cancers, but this national average aggregates all gastric cancer subtypes and can obscure outcomes for gastric adenocarcinoma, the most common and lethal histology. In analyses of seven high-risk states comprising more than half of the US Asian and Hispanic American population, the overall 5-year survival for non-cardia gastric adenocarcinoma—the subtype most strongly associated with *H. pylori*—was substantially lower, in the low-20% range [26]. By contrast, survival is substantially higher in Japan and Korea, where population-based screening and infection control have been implemented for decades [27]. These differences are unlikely to be fully explained by tumor biology alone; they reflect prevention infrastructures never designed to accommodate migration-related heterogeneity in risk.

The consequences of this omission are most apparent at diagnosis. Multiple analyses demonstrate that gastric cancer is disproportionately detected at advanced stages among East Asian, Hispanic, and Eastern European immigrant populations in the United States [14, 25]. These disparities mirror global inequities in early detection but are amplified by US-specific structural barriers, including limited insurance coverage, inconsistent reimbursement for *H. pylori* testing, and low provider awareness of migration-informed risk factors [13, 28]. Federal research investment in gastric cancer remains among the lowest relative to mortality burden, reinforcing a cycle in which prevention gaps persist largely unexamined [29].

Recent policy developments suggest that this pattern is not inevitable. In August 2025, bipartisan lawmakers introduced the Stomach Cancer Prevention and Early Detection Act, the first federal legislation aimed at expanding *H. pylori* testing, improving surveillance in high-risk communities, and supporting research on infection-related gastric cancer [30]. Although still early in its legislative trajectory, the Act signals growing recognition that US prevention strategies must evolve to reflect migration-linked risk. Its introduction underscores a central point: structural omission is a consequence of design choices, not biological fate, and can be corrected when prevention frameworks are aligned with population reality.

Even where population-based *H. pylori* screening is considered, including in light of emerging international recommendations supporting its feasibility and cost-effectiveness [31], underlying heterogeneity in risk related to nativity and early-life exposure may remain relevant, particularly in diverse populations, and may inform how such strategies are implemented and prioritized. In this context, migration-informed approaches are applicable across both primary and secondary prevention, rather than being limited to a single intervention model.

## Toward a Migration-Responsive Cancer Equity Framework

Cancer-equity frameworks in the United States were developed in an earlier era and were not designed with large-scale global mobility in mind. As migration increasingly shapes population composition and disease distribution, equity approaches may need to expand to better reflect how cancer risk is acquired, persists, and is expressed across the life course. A migration-responsive framework can be understood as a pragmatic extension that integrates nativity, early-life exposure, and transnational determinants into how risk is measured, interpreted, and addressed across surveillance, clinical care, research, and policy. This approach reflects a distinct pathway for incorporating risk into prevention, grounded in exposure history and population-level epidemiologic patterns, rather than in individualized genetic susceptibility, and is intended as a selective extension of existing prevention frameworks rather than a separate model of risk-based screening.

At the foundation, surveillance systems represent a critical opportunity for refinement. Current US platforms, including SEER, NHANES, BRFSS, and state cancer registries, routinely capture race and ethnicity but rarely include migration-relevant variables such as country of birth, parental nativity, or age at migration [21, 32]. Even limited incorporation of these elements could reveal clinically meaningful heterogeneity that remains obscured within broad racial categories and enable more precise identification of high-risk populations [20, 21]. Without such data, disparities linked to migration remain difficult to detect and, consequently, challenging to address. Implementation of migration-informed approaches may face practical challenges, including limitations in data collection, variability in documentation of migration history, and policy and resource constraints that shape prevention priorities.

Clinical prevention represents a second, immediately actionable domain, where modest and pragmatic adaptations may help incorporate migration-related risk into existing frameworks. Migration-linked risk can be operationalized through targeted, feasible measures such as electronic health record prompts for *H. pylori* testing based on country of origin, culturally and linguistically concordant educational materials, partnerships with community organizations to expand access, and reimbursement structures that recognize infection eradication as preventive care [6, 22–24]. Community health centers and federally qualified health clinics, already central to care for many immigrant populations, are well positioned to serve as hubs for risk-based prevention, building on established models from hepatitis B and tuberculosis control. These approaches are intended as incremental extensions of existing systems rather than a shift toward fully individualized screening models.

In parallel, migration-responsive prevention can extend beyond infection detection to earlier identification of premalignant gastric conditions. Atrophic gastritis and gastric intestinal metaplasia, well-established precursors of gastric cancer [33], are highly prevalent in many high-risk immigrant populations and, once identified, can be incorporated into risk-based surveillance strategies. Although major gastroenterology societies increasingly endorse surveillance for these lesions, recommendations remain inconsistently implemented in US practice, and clinician awareness varies. Strengthening education around premalignant gastric pathology and embedding surveillance pathways into routine care would support a shift from episodic detection toward more longitudinal prevention for populations with persistent, exposure-linked risk [23].

Research priorities similarly warrant recalibration. Despite clear epidemiologic signals, cancers that disproportionately affect immigrant and diaspora communities, such as gastric, liver, and nasopharyngeal carcinoma, remain underrepresented in national funding portfolios [13, 16, 29]. Migration-responsive research agendas that emphasize exposure-based risk modeling, community-engaged prevention approaches, and training in migration-informed epidemiology would better align scientific investment with contemporary patterns of disease burden.

Finally, domestic cancer-control policy can benefit from closer engagement with global evidence. Experiences from countries such as Japan and Korea demonstrate that population-based endoscopic screening and *H. pylori* eradication can reduce gastric cancer mortality [34, 35]. The World Health Organization and IARC similarly emphasize infection control as a cornerstone of cancer prevention. Thoughtful adaptation of these principles, rather than wholesale transplantation, offers a pathway for low-incidence but high-diversity settings to refine prevention strategies while preserving existing equity commitments.

A migration-responsive framework does not replace traditional equity models; it extends their reach. By incorporating migration-related determinants into surveillance, prevention, research, and policy, health systems can address persistent blind spots and move toward a more complete understanding of how cancer risk is distributed in an interconnected world [1, 22, 30]. Given the logistical and resource constraints that shape screening implementation, integration of migration-related risk is best understood as selective, incremental, and context-dependent.

## Conclusion

Health equity in the twenty-first century can no longer be framed solely within national boundaries. As migration reshapes the distribution of preventable cancers, equity models grounded only in domestic history and race-based categories risk overlooking populations whose risk is shaped by early-life exposure and transnational determinants. In such settings, disparities arise not only from historical discrimination but from structural invisibility within contemporary prevention systems. A migration-informed approach does not replace established equity frameworks; it extends them—integrating nativity, early-life exposure, and migration history into surveillance, clinical prevention, research, and policy. Emerging efforts, including the 2025 Stomach Cancer Prevention and Early Detection Act, signal growing recognition that this alignment is both feasible and necessary. Ultimately, incorporating migration into cancer-equity frameworks can be understood as a shift from static notions of disparity toward a dynamic understanding of risk in an interconnected world—ensuring that populations shaped by global patterns of disease are neither overlooked nor left behind.
